# Comparison between transrectal and transperineal prostate biopsy for detection of prostate cancer: a meta-analysis and trial sequential analysis

**DOI:** 10.18632/oncotarget.15056

**Published:** 2017-02-03

**Authors:** Jianxin Xue, Zhiqiang Qin, Hongzhou Cai, Chuanjie Zhang, Xiao Li, Weizhang Xu, Jingyuan Wang, Zicheng Xu, Bin Yu, Ting Xu, Qin Zou

**Affiliations:** ^1^ Department of Urology, The Second Affiliated Hospital of Southeast University, Nanjing, 210003, China; ^2^ Department of Urology, The First Affiliated Hospital of Nanjing Medical University, Nanjing, 210029, China; ^3^ Department of Urologic Surgery, The Affiliated Cancer Hospital of Jiangsu Province of Nanjing Medical University, Nanjing, 210009, China; ^4^ First Clinical Medical College of Nanjing Medical University, Nanjing, 210029, China; ^5^ Department of Thoracic Surgery, Nanjing Medical University Affiliated Cancer Hospital, Jiangsu Key Laboratory of Molecular and Translational Cancer Research, Cancer Institute of Jiangsu Province, Nanjing, 210009, China; ^6^ Department of Gastrointestinal Oncology, Key Laboratory of Carcinogenesis and Translational Research (Ministry of Education), Peking University Cancer Hospital and Institute, Beijing 100142, China

**Keywords:** transperineal, transrectal, prostate biopsy, prostate cancer, meta-analysis

## Abstract

To systematically assess the efficacy and complications of transrectal (TR) versus transperineal (TP) prostate biopsy in the detection of prostate cancer (PCa). A meta-analysis was performed by searching the databases Pubmed, Embase and Web of science for the relevant available studies until September 1st, 2016, and thirteen studies met the inclusion criteria. The pooled odds ratios with 95% confidence intervals were calculated to evaluate the differences of TR and TP groups in PCa detection rate. Then, trial sequential analysis was performed to reduce the risk of type I error and estimated whether the evidence of the results was reliable. Overall, this meta-analysis included a total of 4280 patients, who had been accrued between April 2000 and Aug 2014 and randomly divided into TR group and TP group. Prostate biopsies included sextant, extensive and saturation biopsy procedures. Patients who received TP prostate biopsy had no significant improvement in PCa detection rate, comparing TR group. Moreover, when comparing TR and TP studies, no significant difference was found in abnormal DRE findings, serum PSA level measurement, Gleason score, prostate volume. Besides, this meta-analysis showed no obvious differences between these two groups in terms of relevant complications. Therefore, this meta-analysis revealed that no significant differences were found in PCa detection rate between TP and TR approaches for prostate biopsy. However, with regard to pain relief and additional anesthesia, TR prostate needle biopsy was relatively preferable, compared to TP prostate biopsy.

## INTRODUCTION

Prostate cancer (PCa) was the most frequently diagnosed malignancy among the male population in the western countries [1]. Although the data from the American Society showed that the estimated 5-year survival rate was 98.9%, PCa remained second leading cause of cancer-related death among men in USA [1, 2]. Therefore, there is an urgent need for a better diagnostic technology for early detection of PCa. The elevated serum prostate-specific antigen (PSA) level measurement, abnormal digital rectal examination (DRE) finding and transrectal ultrasonography (TRUS), as widely opportunistic screening tools, have been widely used to diagnose patients at a high risk of PCa [3]. Moreover, since the systematic sextant prostate biopsy was firstly introduced to detect PCa by Hodge et al. [4] by TRUS guidance, prostate biopsy had become a widely-accepted and routinely-performed technology to detect PCa [5]. However, the optimal biopsy strategy for PCa detection remained to be completely defined.

Transperineal (TP) biopsy and Transrectal (TR) biopsy are the two primary approaches in the detection of PCa by obtaining prostate tissue specimen. There are a lot of differences between the two, such as puncture site, puncture route and the TRUS transducer. Currently, lack of standardization lies in the differences of TP and TR approaches in the rates of PCa detection. In TR biopsy, the needle punctures through the anterior rectal wall under the guidance of an end-fire transducer. But in TP biopsy, the needle punctures through skin of perineum under the guidance of a bi-planar transducer [6]. Early studies have reported that TP biopsy was superior to TR biopsy with regard to detecting PCa [7]. That was probably because that TP biopsy was targeted to the lateral, apico-dorsal peripheral and transition zones in the routine biopsy might increase PCa detection rates [8–11]. In addition, previous studies had recommended an increase in the number of biopsy cores [12–14]. However, the number of biopsies required for optimizing the diagnosis of PCa was still controversial. With the increased number of biopsy cores, more and more studies involved deemed that they were equivalent, compared to TP and TR approaches in PCa detection rate [15, 16]. Besides, with respect to the associated complications, it seemed that no significant differences were detected between the two.

To date, several clinical randomized trials have investigated the detailed efficacy and complications in the patients with a high risk of PCa in the comparison between TR and TP biopsy. However, the results remained inconsistent or even contradictory. In addition, lack of further researches in various relevant complications systematically illustrated comprehensive understanding of the differences about TR and TP approaches in patients with high-risk PCa in previous meta-analyses [17]. Hence, in order to compare PCa diagnostic accuracy and complications of TP and TR biopsies, an updated meta-analysis was conducted by including all individual patient data from eligible studies to identify this statistical evidence. Furthermore, trial sequential analyses (TSA) were for the first time used to clarify whether the evidence for the results was sufficient.

## RESULTS

### Studies characteristics

**Figure 1:**
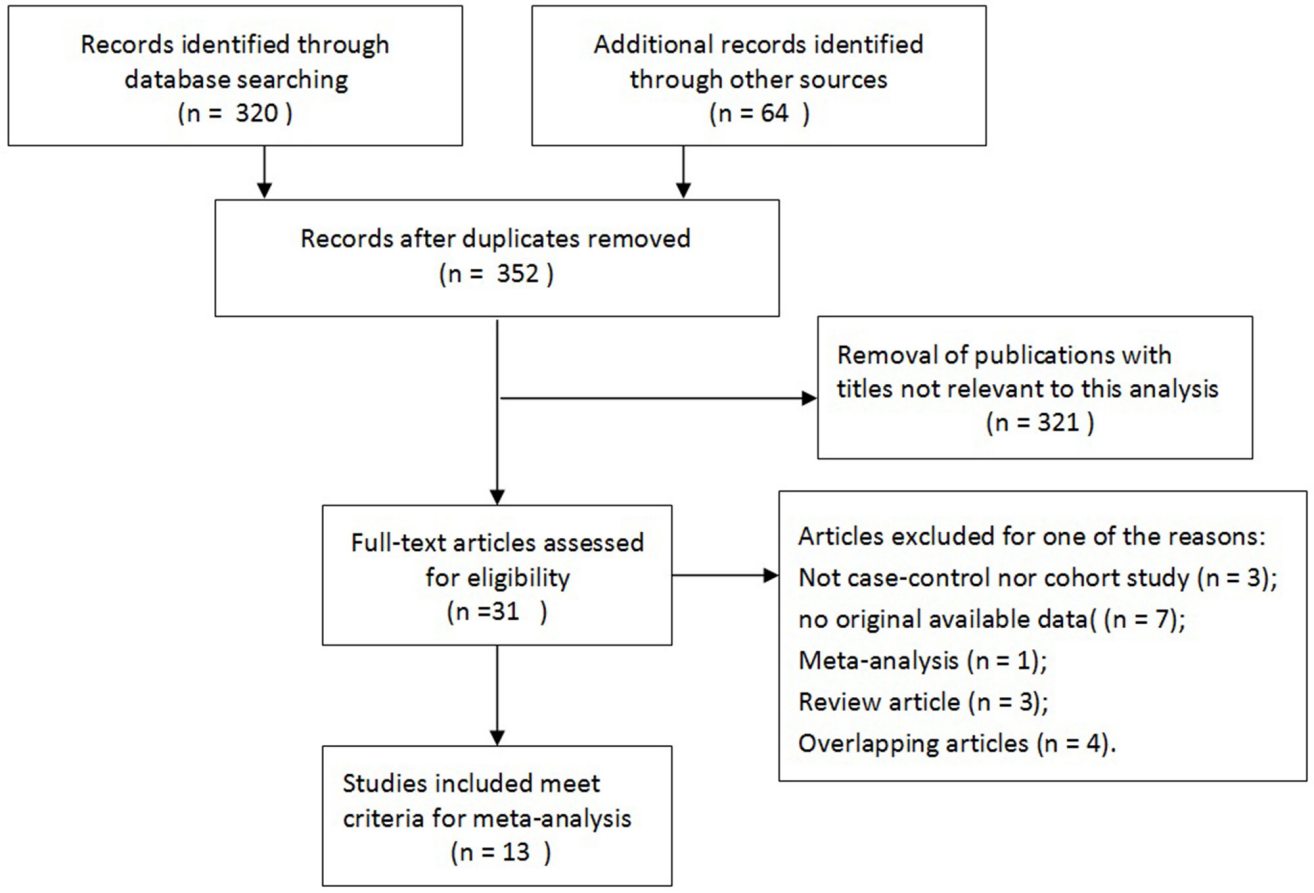
Flow diagram of literature search and selection process

A total of thirteen studies including 4280 patients met the inclusion criteria and were involved in the present meta-analysis [6, 15, 18–25], which had been accrued between April 2000 and Aug 2014. All of the baseline characteristics of the studies involved are comprehensively listed in Table 1. Besides, patients were randomly divided into TR group and TP group. In this meta-analysis, these trials were divided into three groups: the sextant biopsy group (four studies) [18, 22, 25], extensive biopsy group (seven studies) [15, 19–21, 23] and saturation biopsy group (one study) [24]. In addition, one study by Guo et al. [6] mentioned two kinds of biopsy groups including sextant biopsy and extensive biopsy approaches. Figure 1 shows the flowchart of literature search and screening process.

**Table 1:** Characteristics of individual studies included in the meta-analysis

Year	Surname	Country	Ethnicity	Patients (n)		Mean age (years)		PSA (ng/ml)		Total prostate volume (mL)		No. of cores		Biopsy group	Design
				TR group	TP group	TR group	TP group	TR group	TP group	TR group	TP group	TR group	TP group		
2015	Guo	China	Asian	166	173	67.35±7.28	67.18±6.76	10.48 (6.2–69.0)	8.81(3.6–56.0)	45.9(20.0–98.0)	47.2 (12.9–97.7)	12 or 8†	12 or 8†	Mixed	RCT
2014	Cerruto	Italy	Caucasian	54	54	67.30±8.05	66.50±8.87	12.36±39.65	15.95±41.04	61.49±33.39	56.29±31.33	14	14	Extensive	PCS
2014	Yuan	China	Asian	97	59	66.1	65.4	19.7 (7.8–362.0)	21.2(8.9–235.0)	35.8(19.8–93.6)	33.7 (21.3–87.5)	6 + 2‡	6 + 2‡	Sextant	CCS
2014	Miano	Italy	Caucasian	255	278	64.0±6.2	64.6±5.8	8.6±4.1	8.6±5.3	42.3±16.7	38.9±29.7	12	12	Extensive	CCS
2012	Hossack	Australia	Caucasian	718	414	NM	NM	NM	NM	50.8±18.4	52.4±17.2	12	12	Extensive	CCS
2011	Abdollah	Italy	Caucasian	140	140	66.2 (47.6–82.1)	66.4 (52.0– 79.0)	9.7 (2.1–26.2)	10 (0.9–31.5)	65.4 (15.0–193.0)	62.3 (17.0–198.0)	24	24	Saturation	CCS
2009	Chae	Korea	Asian	100	100	66.6±9.03	64.4±9.76	13.8±20.39	23.1±103.67	NM	NM	12	12	Extensive	RCT
2008	Takenaka	Japan	Asian	100	100	72.1±7.42	71.1±7.53	19.6±43.2	17.1±30.1	37.2±19.7	34.5±18.9	12	12	Extensive	RCT
2007	Kawakami	Japan	Asian	231	243	68 (64–73)	68 (64–72)	7.7 (5.3–12)	7.6 (5.3–12)	29 (23–42)	29 (22–40)	12	14	Extensive	CCS
2008	Hara	Japan	Asian	120	126	71.7±7.55	71.0±7.29	8.48±3.90	8.34±3.44	36.0±17.1	33.2±15.2	12	12	Extensive	RCT
2005	Watanabe	Japan	Asian	161	166	72.5 (41–98)	10.3 (0.6–460.0)	NM	NM	6	6	Sextant	CCS
2005	Miller	Australia	Caucasian	103	75	66.9±1.5	69.5±1.4	58.5±38.0	19.1±5.4	NM	NM	6	6	Sextant	CCS
2003	Emiliozzi	Italy	Caucasian	107	68 (52–88)	8.2(4.1–240)	NM	NM	6	6	Sextant	CCS

NM: not mentioned;

†:12 cores were sampled from the prostate with volume > 50ml, whereas 8 cores from prostate with volume < 50 ml;

‡:On the basis of the 6 needle prostate puncture, Plus 1 needles on both sides of the prostatic peripheral zone.

### Quantitative synthesis results

Overall, patients who received TP prostate biopsy had no significant improvement in PCa detection rate, comparing TR group. Moreover, when comparing TR and TP studies, no significant differences were found in abnormal DRE findings, serum PSA level measurement, Gleason score, prostate volume. Besides, this meta-analysis showed no obvious differences between these two groups in terms of related complications.

#### prostate cancer detection rate

**Figure 2:**
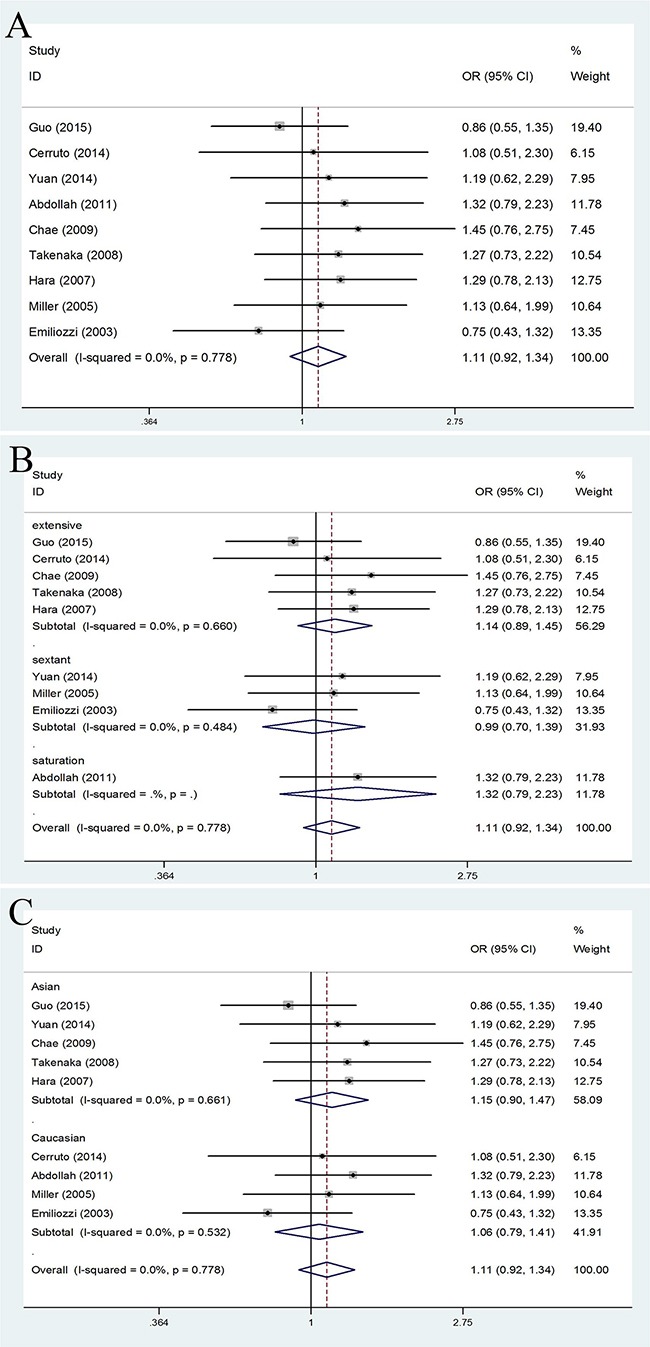
Forest plots of PCa detection rate compared with TR and TP prostate biopsy **A**. Forest plots of efficacy of TR versus TP prostate biopsy in the PCa detection rate; **B**. Forest plots of subgroup analysis by number of biopsy cores in the PCa detection rate compared with the two; **C**. Forest plots of subgroup analysis by ethnicity in the PCa detection rate compared with the two.

No significantly differences were found in PCa detection rate of TR versus TP prostate biopsy (OR = 1.11, 95% CI = 0.92–1.34). (Figure 2A). When the studies were stratified by number of biopsy cores, the results were still no significantly differences whether in extensive biopsy group (OR = 1.14, 95% CI = 0.89–1.45), sextant biopsy group (OR = 0.99, 95% CI = 0.70–1.39) or saturation biopsy group (OR = 1.32, 95% CI = 0.79–2.23). (Figure 2B) Moreover, in the stratified analysis by ethnicity, no significant results were detected in both Asian population (OR = 1.15, 95% CI = 0.90–1.47) and Caucasian population(OR = 1.06, 95% CI = 0.79–1.41). (Figure 2C)

#### abnormal DRE findings

**Figure 3:**
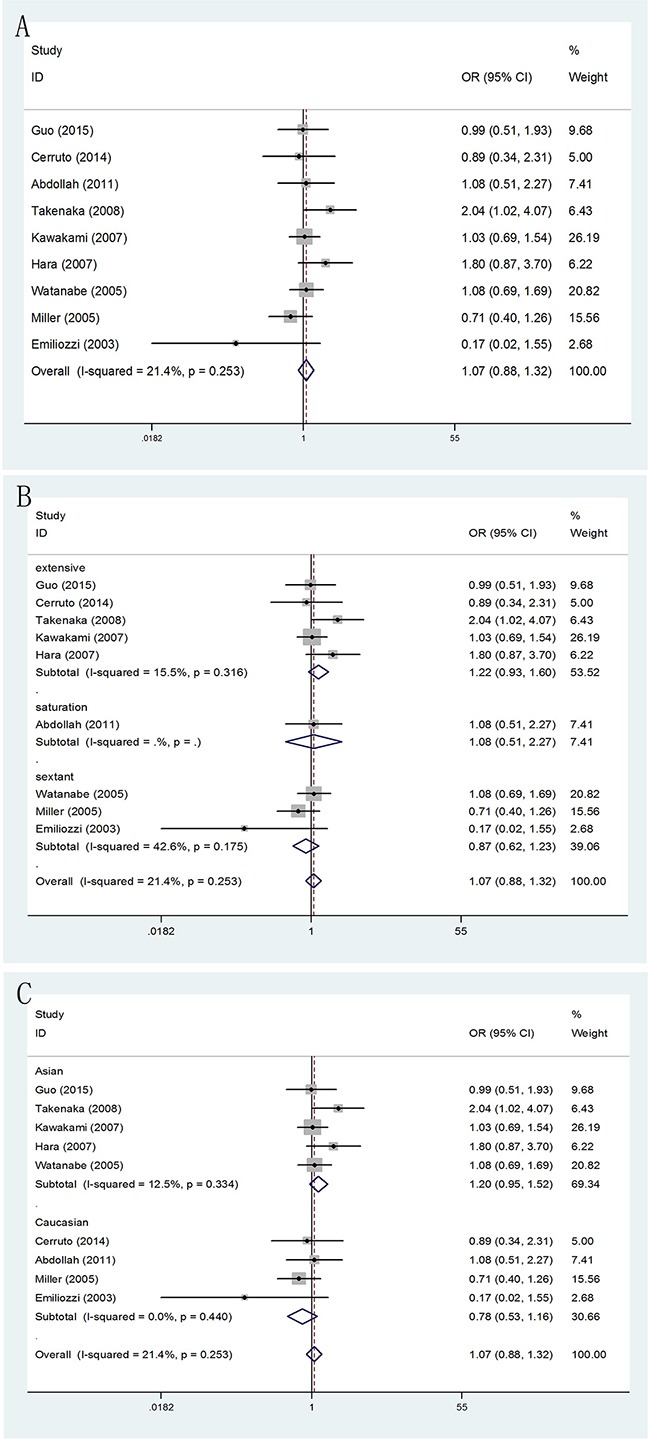
Forest plots of abnormal DRE findings compared with TR and TP prostate biopsy **A**. Forest plots of efficacy of TR versus TP prostate biopsy in abnormal DRE findings; **B**. Forest plots of subgroup analysis by number of biopsy cores in abnormal DRE findings compared with the two; **C**. Forest plots of subgroup analysis by ethnicity in abnormal DRE findings compared with the two.

Meanwhile, patients who received TP prostate biopsy had no obvious improvement in abnormal DRE findings, compared with TR prostate biopsy group (OR = 1.07, 95% CI = 0.88–1.32). (Figure 3A) In the subgroup analysis by number of biopsy cores, the results were no significant in extensive biopsy group (OR = 1.22, 95% CI = 0.93–1.60), in sextant biopsy group (OR = 0.87, 95% CI = 0.62–1.23) and in saturation biopsy group (OR = 1.08, 95% CI = 0.51–2.27). (Figure 3B) Besides, when the studies were stratified by ethnicity, we detected no significantly differences in both Asian population (OR = 1.20, 95% CI = 0.95–1.52) and Caucasian population (OR = 0.78, 95% CI = 0.53–1.16). (Figure 3C)

#### serum prostate-specific antigen (PSA) level measurement

**Figure 4:**
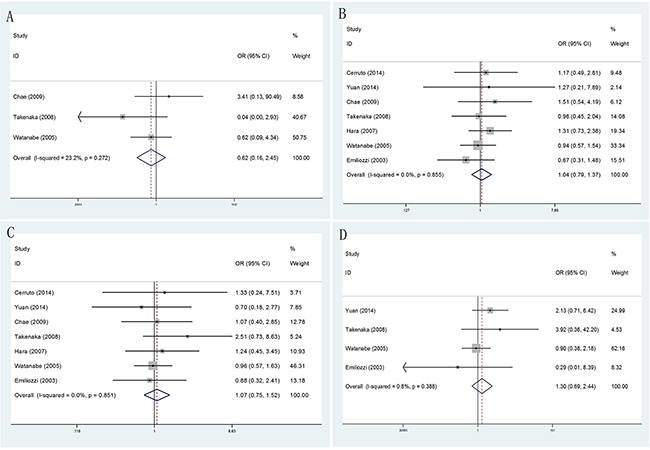
Forest plots of different PSA levels compared with TR and TP prostate biopsy **A**. Forest plots of efficacy of TR versus TP prostate biopsy in PSA≤4ngml^−1^; **B**. Forest plots of efficacy of TR versus TP prostate biopsy in 4ngml^−1^<PSA≤10 ng ml^−1^; **C**. Forest plots of efficacy of TR versus TP prostate biopsy in 10ngml^−1^<PSA≤20ngml^−1^; **D**. Forest plots of efficacy of TR versus TP prostate biopsy in PSA>20ngml^−1^.

**Figure 5:**
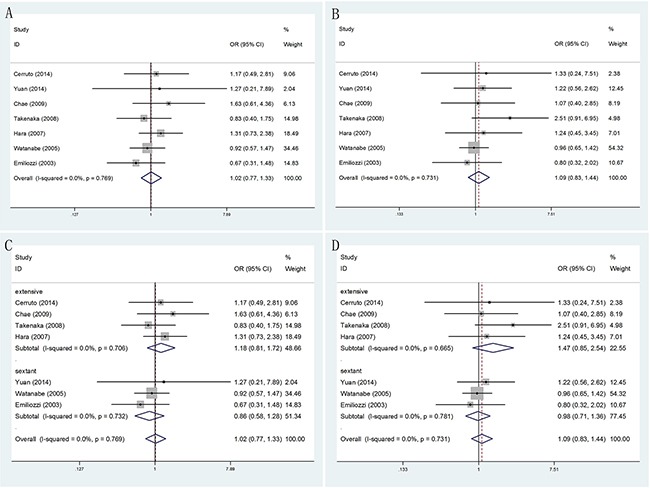
Forest plots of different PSA levels (PSA≤10ngml^−1^ and PSA>10ngml^−1^) compared with TR and TP prostate biopsy **A**. Forest plots of efficacy of TR versus TP prostate biopsy in PSA≤10ngml^−1^; **B**. Forest plots of efficacy of TR versus TP prostate biopsy in PSA>10ngml^−1^; **C**. Forest plots of subgroup analysis by number of biopsy cores in PSA≤10ngml^−1^ compared with the two; **D**. Forest plots of subgroup analysis by number of biopsy cores in PSA>10ngml^−1^ compared with the two.

According to different PSA levels (PSA≤4ngml^−1^, 4ngml^−1^<PSA≤10ngml^−1^, 10ngml^−1^<PSA≤20ngml^−1^ and PSA>20ngml^−1^), no significant results were detected in any subgroup (Figure 4). Furthermore, through subgroup analyses by different serum PSA level measurement (PSA≤10ngml^−1^ and PSA>10ngml^−1^), the result showed no obvious differences between PSA≤10ngml^−1^ (OR = 1.02, 95% CI = 0.77–1.33) (Figure 5A) and PSA>10ngml^−1^ (OR = 1.09, 95% CI = 0.83–1.44) (Figure 5B). In addition, whether in PSA≤10ngml^−1^ group (Figure 5C) and PSA>10ngml^−1^ group (Figure 5D), no significant results were detected in both extensive biopsy group and sextant biopsy group in the stratified analysis by number of biopsy cores.

#### Gleason score

**Figure 6:**
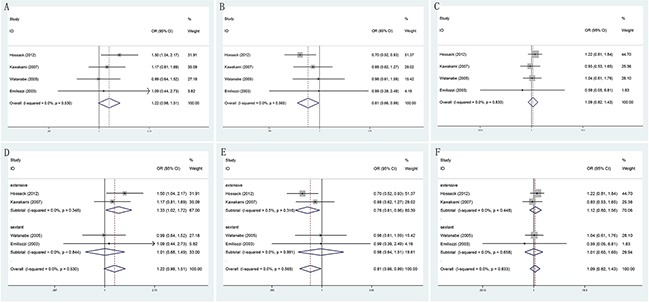
Forest plots of biopsy Gleason score compared with TR and TP prostate biopsy **A**. Forest plots of efficacy of TR versus TP prostate biopsy in Gleason score ≤6; **B**. Forest plots of efficacy of TR versus TP prostate biopsy in Gleason score =7; **C**. Forest plots of efficacy of TR versus TP prostate biopsy in Gleason score ≥8; **D**. Forest plots of subgroup analysis by number of biopsy cores in Gleason score ≤6 compared with the two; **E**. Forest plots of subgroup analysis by number of biopsy cores in Gleason score =7 compared with the two; **F**. Forest plots of subgroup analysis by number of biopsy cores in Gleason score ≥8 compared with the two.

When comparing TR and TP biopsy groups in biopsy Gleason score ≤6 (OR = 1.22, 95% CI = 0.98–1.51) and ≥8 (OR = 1.09, 95% CI = 0.82–1.43), there were no statistically significant differences in two groups of patients. Besides, statistically significant was found only among biopsy Gleason score =7 (OR = 0.81, 95% CI = 0.66–0.99). Moreover, in the extensive biopsy group, subgroup analysis showed that significant results increased risk in TR prostate biopsy group in Gleason score ≤6 (OR = 1.33, 95% CI = 1.02–1.72). However, when Gleason score was equal to 7 (OR = 0.76, 95% CI = 0.61–0.96), we found statistically significant differences decreased risk in TR prostate biopsy group. Meanwhile, no statistically significant differences were found in any other groups (Figure 6).

#### Prostate volume

**Figure 7:**
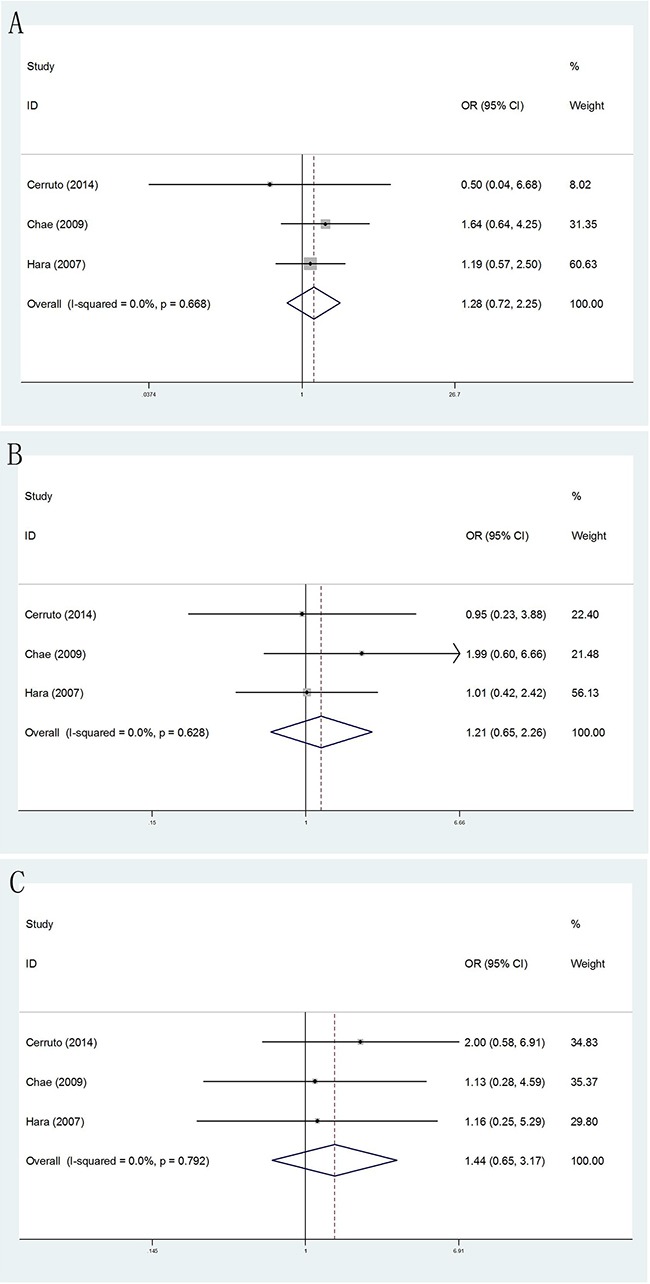
Forest plots of prostate volume compared with TR and TP prostate biopsy **A**. Forest plots of efficacy of TR versus TP prostate biopsy in prostate volume <30ml; **B**. Forest plots of efficacy of TR versus TP prostate biopsy in 30 ml≤prostate volume ≤50 ml **D**; **C**. Forest plots of efficacy of TR versus TP prostate biopsy in prostate volume ≥50 ml.

No matter how size of prostate volume, we did not still find there were significantly differences when comparing TR and TP biopsy approaches. (Figure 7)

#### Complications

In current meta-analysis, results of relevant complications comparing TR versus TP biopsies were showed in Table 2. Outcomes showed that there were no significantly differences in all complications involved, including hematuria (20.6% vs17.1%; OR = 1.14, 95% CI = 0.85,1.53), rectal bleeding (10.2% vs1.5%; OR = 4.49, 95% CI = 0.51,39.22), hematospermia (0.7% vs1.2%; OR = 0.59, 95% CI = 0.14,2.47), sepsis (0.4% vs0.4%; OR = 0.93, 95% CI = 0.15,5.82), fever (1.6% vs0.2%; OR = 5.46, 95% CI = 0.84,35.58), urinary retention (3.8% vs2.4%; OR = 1.39, 95% CI = 0.57,3.37), vasovagal event (1.5% vs0.9%; OR = 1.57, 95% CI = 0.51,4.82), post-dural puncture headache (0.0% vs3.1%; OR = 0.03, 95% CI = 0.00,2.44).

**Table 2:** Outcomes of Related complications comparing transperineal and transrectal prostate biopsy

Complications	Trials	TR group	TP group	Heterogeneity		OR (95% CI)	P-value
				P-value	I2 (%)		
Hematuria	6	143/694	108/633	0.817	0	1.14 (0.85,1.53)	0.366
Rectal bleeding	7	76/748	10/687	0.001	74.0	4.49 (0.51,39.22)	0.174
Hematospermia	4	3/436	5/407	0.441	0	0.59 (0.14,2.47)	0.475
Sepsis	4	2/497	2/474	0.765	0	0.93 (0.15,5.82)	0.936
Fever	4	7/435	1/447	0.789	0	5.46 (0.84,35.58)	0.073
Urinary retention	4	14/371	8/339	0.907	0	1.39 (0.57,3.37)	0.465
Vasovagal event	5	8/551	5/528	0.872	0	1.57 (0.51,4.82)	0.432
Post-dural puncture headache	2	0/220	7/226	0.842	0	0.03 (0.00,2.44)	0.117

### Test of heterogeneity

Heterogeneity between studies was observed in PCa detection rate of TR and TP approaches respectively, but the heterogeneity was decreased through subgroup analyses. In addition, there was no prominent heterogeneity (*P* = 0.778), and the pooled OR for PCa detection rate was performed using Fixed-effort model.

### Sensitivity analysis

Sensitivity analysis was utilized to detect the influence of each study on the pooled OR by repeating the meta-analysis while omitting one single study each time. The sensitivity analysis for the results of TR and TP approaches in PCa detection rate demonstrated that no individual study affected the pooled OR significantly. Thus, sensitivity analysis showed that our results were statistically robust.

### Publication bias

**Figure 8:**
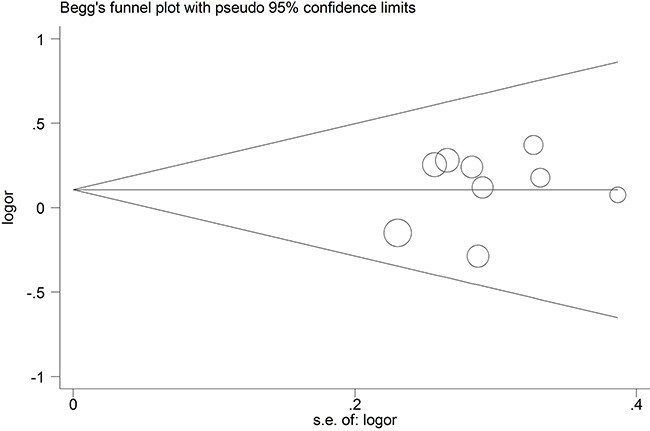
Begg's funnel plot of publication bias test in the PCa detection rate compared with TR and TP prostate biopsy

The Begg's funnel plot was applied to assess the publication bias of the literature, and the shapes of them seemed no evidence of obviously asymmetrical, indicating no significant publication bias, which was also confirmed according to funnel plot (Begg's test, P = 0.835; Egger's test, P = 0.606). (Figure 8) Therefore, the overall outcomes indicated that our findings were reliable.

### Trial sequential analysis results

**Figure 9:**
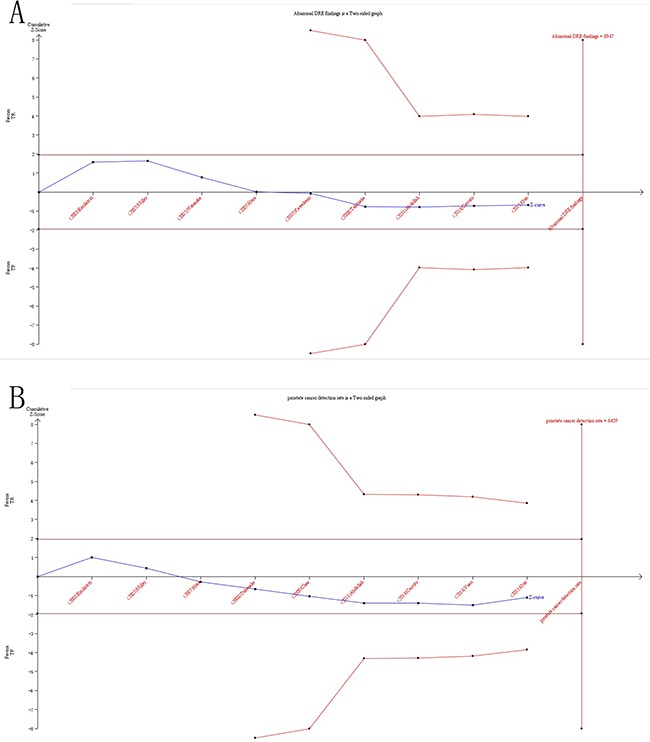
Trial sequential analysis of efficacy of TR versus TP prostate biopsy **A**. TSA of PCa detection rate compared with TR and TP prostate biopsy; **B**. TSA of abnormal DRE findings compared with TR and TP prostate biopsy. The required information size was calculated based on a two side α= 5%, β= 20% (power 80%), and a relative risk reduction of 20%.

In our current study, the cumulative Z-curve (the blue line) did not exceed the information size (vertical red line) (Figure 9) in both PCa detection rate and abnormal DRE findings, suggesting in-sufficient evidence of efficacy of TR versus TP biopsy. Therefore, our results need to be further checked with a sufficiently large number of participants to certify the previously reported differences in well-designed studies.

## DISCUSSION

With the widespread clinical use of opportunistic screening tools, such as PSA, DRE and TRUS, prostate biopsy has become one of the most commonly performed urological technologies. Systematic biopsy of the prostate has been considered as the golden standard for the diagnosis of PCa, confirming the grading of PCa and stratifying tumor aggressiveness. The reason for this is because that the definite detection of PCa depends on the histopathological verification of cancer in prostate biopsy cores or surgical specimens. Likewise, in recent years, the number of cores taken on baseline biopsy has increased from six to twelve, while in the repeat biopsy setting more extended and saturation protocols are regularly applied. The two major techniques for prostate biopsy are the TR and TP biopsy respectively. Though the two approaches seem to have the same PCa detection rate and overall complication rate, it is interesting to note that TR biopsy is more popular globally [26]. That is because that compared to TP approach, TR prostate biopsy have some advantages including more time-saving, relatively simple operation as well as the non-essential for high-grade anesthesia. Therefore, Either American Urology Association and European Association of Urology recommends that TR biopsy is used as the most common method, while TP biopsy is a useful alternative [29].

Recently, increasing relevant studies researched clinical efficacy and complications of TR and TP prostate biopsy in the detection of PCa. To the best of our knowledge, this was an updated meta-analysis that systematically and comprehensively investigated the efficacy and adverse events in two kinds of prostate biopsy approaches, in order to elucidate such differences. Nevertheless, the outcomes remained inconsistent or unclear. The conflict among them might partially own to the relatively small sample size of individual studies, the different ethnicities and the possible limited effect of individual patient data in prostate biopsy. All these contributed to the limited statistical power in the published studies. Moreover, TSA was adopted to test for the first time in the present meta-analyses. Therefore, we needed a better method to assess the efficacy and complications of TR and TP prostate biopsy in the detection of PCa. On the one hand, further researches in different stratified analysis were necessary in these meta-analyses. On the other hand, for the first time, we applied TSA to reduce the risk of type I error and testify whether the evidence of our results was sufficient. Moreover, quiet a few meta-analyses explored results of TR and TP prostate biopsy [17], but the results in early articles involved differed a lot. What's more, lack of further researches by different stratified analysis in these meta-analyses prevented comprehensive understanding of the disparity and different between two groups. Furthermore, additional studies about such distinction had been published since the previous meta-analysis, which might generate great influence on the results. All these factors contributed to the development of the current meta-analysis.

This meta-analysis of individual patient data confirmed that, compared with TR approach, using TR approach did not markedly increase rates of PCa detection, and relieve adverse events in patients with prostate biopsy. Meta-analysis as a powerful tool could provide more reliable results than a single study especially in explaining controversial conclusions [28]. As a consequence, we took advantage of meta-analysis to clarify which was an advanced method in the diagnosis of PCa. To the best of our knowledge, meta-analysis could also provide the most comprehensive information by different subgroup analysis. In addition, in the stratified analysis by number of biopsy cores and ethnicity, the results showed there were no significantly differences in both two groups. Thus, findings of our current meta-analysis suggested that TP prostate biopsy was no superior to TR prostate biopsy.

Patients who received TP prostate biopsy had no improvement in PCa detection rate, comparing TR group. Likewise, in the stratified analysis by number of biopsy cores and ethnicity, no significant results was still detected. Moreover, to further compare the differences between the two methods, we performed statistical analysis in abnormal DRE findings, serum PSA level measurement, Gleason score, prostate volume. But, the results of meta-analysis showed that no significantly differences were found except when Gleason score =7 or Gleason score ≤6 in the extensive biopsy group. It was likely that limited number of trials and the insufficient data of articles included might result in such conclusions. Therefore, further high-quality researches were needed to confirm these findings in subsequent years.

In current meta-analysis, the results confirmed that no significantly differences in all complications were detected comparing TR versus TP prostate biopsy, including hematuria, rectal bleeding, hematospermia, sepsis, fever, urinary retention, vasovagal event and post-dural puncture headache. In a recent review, a higher risk of infection was arisen in TR biopsy because the faecal carriage bacteria can easily enter the blood from puncture sites [29]. Besides, compared with TP group, rectal bleeding and infection-related complications were more frequently observed in the TR group. Thus, they raised the risk of major complications in the TR group. Though the major complications appeared to be rare, sometimes they might be also life-threatening. From another aspect, previous studies suggested that some shortcomings of TP prostate biopsy including consumption of longer time, training and financial constraints, relatively higher rate of sampling failure as well as the need for additional high-grade anesthesia [30]. Indeed, the above-mentioned factors might limit the use of TP approach; however, as far as we know, no previous studies have been carried out to compare TP group with TR group on the above-mentioned factors in prostate biopsy directly. In addition, according to our experience, complications of rectal bleeding or infection in TP group could relatively decrease because of its uniqueness in puncture route that rectum was not involved. However, in present meta-analysis there were no significant differences in rectal bleeding, fever and sepsis between these two groups. Moreover, specifically, patients with increased risk of rectal bleeding or infection suffered from disease, such as known hemorrhoids, antibiotic resistance or some other situations, TP approach might be a safer alternative. Hence, further studies were needed to clarify this point. Nevertheless, our analysis indicated that the profile of complications between TR versus TP biopsy was equivalent, and all the adverse effects were tolerable and manageable.

TSA is a powerful and useful approach in the goal of summarizing evidence and provided the required information size in meta-analyses [31, 32]. In order to reduce the risk of type I error and estimate whether further trials are needed, TSA is introduced to calculate the required information size for the meta-analysis with the adaptation of monitoring boundaries [33]. However, the meta-analyses not reaching the required sample size are analyzed with trial sequential monitoring boundaries, which is similar to interim monitoring boundaries in a single trial [32, 34–36]. Compare to the traditional meta-analysis, TSA show the potential to be more reliable. If the sufficient information size is not reached, false positive results are early eliminated due to random errors and repeated significance testing in meta-analyses [32, 34, 37]. Besides, when reliable evidence is obtained by TSA, other researchers can stop implementation of remainder studies. Otherwise, this makes it necessary to re-estimate the additional number of patients required to obtain reliable results in the meta-analyses, thereby guiding experimenter in subsequent trials [38, 39]. In the present meta-analysis, the number of cases and controls included were less than the required information size, which meant that our results needed to be further firm evidence of effect.

To a certain extent, some limitations in our meta-analysis should be taken into consideration when interpreting the data. Firstly, with limiting numbers of published studies and insufficient number of patients, the results were based on unadjusted estimates. As a consequence, inclusion criteria about data of each patient in previous articles was difference a lot. So as to the relatively high heterogeneity, which could be reduced by subgroup analysis. Secondly, many factors could affect the accuracy of prostate puncture, such as different puncture site, the number of cores in different zones of the prostate and the proficiency of a particular physician, but these were not considered in our subgroup analysis. Exploring more better puncture strategy was required by more researches in the future. In addition, in the stratified analyses, sample size of some subgroups was relatively small, without enough statistical power to explore the efficacy of TR versus TP biopsy. What's more, no available data of some adverse events in TR versus TP biopsy were not assessed in all included trials. Thus, further exploration in these complications might be conducted in subsequent years. Last but not least, the majority studies used were investigated in Caucasian and Asian population, suggesting analysis result might exist some merits. Hence, to guaranty reliability of our meta-analysis, more researches should focus on the influence of different factors in subsequent articles. Accordingly, it was required that further studies could be performed to elucidate the differences in the effectiveness of prostate puncture in comparison with TR and TP groups if individual data were available.

## CONCLUSION

In summary, the results of the current meta-analysis indicated that no significant differences were found in terms of efficiency and complications between TP and TR approaches for prostatic biopsy. However, with regard to pain relief and additional anesthesia, TR prostate needle biopsy was relatively preferable, compared to TP prostate biopsy. Hence, TP prostate biopsy should be available to urologists as an alternative procedure. As a result, taking into account the current limited data in the included studies, patients with prostatic biopsy, additional high-quality and multicentre studies are needed to further to elaborate such differences in subsequent articles.

## MATERIALS AND METHODS

A comprehensive search was conducted in the databases PubMed, EMBASE and Web of Science for relevant articles, covering all the literatures published until September1st, 2016, and no language restrictions were applied. Through the literature retrieval, combinations of the following sets of keywords were included: “transperineal”, “transrectal” or “prostate biopsy”, “detection” or “diagnosis” and “prostate cancer” or “prostatic neoplasms”. In addition to electronic search original papers, relevant review articles were hand-searched from reference lists of original articles or reviews to find additional eligible studies. Besides, abstract booklets and presentations were also checked from the annual academic conferences. Furthermore, we not only sent emails to the corresponding author to obtain desired information if more data was needed, but also asked participating trialists if they were aware of studies not retrieved by the trial search. Last but not least, if more than one article had been published using the same series of study subjects, only the study with the most recent or complete data was selected.

Articles involved had to meet the following inclusion criteria: (1) Randomized controlled trials, case-control studies and cohort studies were included; (2) The patients were suspicious of PCa who previously underwent prostate biopsy; (3) Efficacy and complications of trails compared with TP and TR prostate biopsies; (4) Sufficient data from the included studies could be extracted. The major exclusion criterion was as follows: (1) No available information or complete data; (2) Non-original research; (3) Patients with a previous history of PCa, acute prostatitis or proven urinary tract infection; (4) Duplicates of previous publication.

### Data extraction

The identified studies were reviewed carefully by two co-authors (Qin ZQ and Li X) independently to determine whether an individual study met inclusion criteria. All data was centrally extracted from the eligible publications and the disagreement was resolved by a discussion with a third reviewer. The following extracted information was recorded in a standardized form and all the data were selected from included articles: year of publication, first author's name, nationality, ethnicity, study design, number of patients, mean age (years) and range, serum concentration of PSA, mean size of total prostate volume, number of biopsy cores and some relevant complications.

### Statistical analysis

The pooled odds ratios (ORs) with 95% confidence intervals (CIs) were utilized to evaluate the strength of differences in TR and TP approaches. Heterogeneity assumption was verified by calculating the Chi-square test and I-square test. The fixed-effects model (Mantel-Haenszel method) and the random-effects model (DerSimonian-Laird method) were respectively used in the meta-analysis. If the presence of heterogeneity was detected, the random-effects model would be conducted. Otherwise, fixed-effects model would be used. Besides, the sources of heterogeneity were explored if there was significant heterogeneity among studies. Thus, subgroup analysis was further carried out by different number of biopsy cores and ethnicity. In addition, sensitivity analysis was performed with the method of appraising the stability of results by omitting one single study each time. Moreover, publication bias was examined by Begg's funnel plot and further checked by Egger's linear regression test between the studies. P values were all two-sided and were considered statistically significant when less than 0.05 [40]. All statistical data were conducted by using Stata software (version 12.0; StataCorp LP, College Station, TX).

### Trial sequential analysis

Outcomes of meta-analysis may result in type I errors due to repetitive testing of accumulated data and are prone to systematic or random errors with sparse data collected [32, 37]. Therefore, TSA was introduced, which estimated the required information size by adjusting threshold for significance level with dispersed data and confirmed more statistical reliability of the data than that of the traditional meta-analysis [37, 41–43]. In the current meta-analysis, TSA was performed by maintaining a 95% CIs, a 20% relative risk reduction, an overall type-I error of 5%, and a statistical test power of 80%, which the required information size was calculated and the trial sequential monitoring boundaries was constructed. If the cumulative Z-curve (the blue line) crossed the trial sequential monitoring boundary (sloping red line) or exceeded the required information size (vertical red line), a significant result had been reached and no further studies were needed. Otherwise, if the cumulative Z-curve did not cross the boundary or the information size required had not been reached, we needed additional clinical trials to reach the adequate information size required to obtain sufficient evidence [32, 35–37, 44]. The trial sequential analysis software (TSA, version 0.9; Copenhagen Trial Unit, Copenhagen, Denmark, 2011) was performed in this study.
